# Human Milk Protein-Derived Bioactive Peptides from In Vitro-Digested Colostrum Exert Antimicrobial Activities against Common Neonatal Pathogens

**DOI:** 10.3390/nu16132040

**Published:** 2024-06-27

**Authors:** Yang Lyu, Bum Jin Kim, Jagdish Suresh Patel, David C. Dallas, Yimin Chen

**Affiliations:** 1 Margaret Ritchie School of Family and Consumer Sciences, University of Idaho, Moscow, ID 83844, USA; ylyu0860@gmail.com; 2 Nutrition Program, College of Health, Oregon State University, Corvallis, OR 97331, USA; bumjin.kim@oregonstate.edu (B.J.K.); dave.dallas@oregonstate.edu (D.C.D.); 3 Department of Chemical and Biological Engineering, University of Idaho, Moscow, ID 83844, USA; jspatel@uidaho.edu

**Keywords:** breast milk, mother’s milk, intestine, antibacterial, lactation, neonate

## Abstract

Human milk reduces risk for necrotizing enterocolitis in preterm infants. Necrotizing enterocolitis occurs in the ileocecal region where thousands of milk protein-derived peptides have been released from digestion. Digestion-released peptides may exert bioactivity, such as antimicrobial and immunomodulatory activities, in the gut. In this study, we applied mass spectrometry-based peptidomics to characterize peptides present in colostrum before and after in vitro digestion. Sequence-based computational modeling was applied to predict peptides with antimicrobial activity. We identified more peptides in undigested samples, yet the abundances were much higher in the digested samples. Heatmapping demonstrated highly different peptide profiles between undigested and digested samples. Four peptides (α_S1_-casein [157–163], α_S1_-casein [157–165], β-casein [153–159] and plasminogen [591–597]) were selected, synthesized and tested against common pathogenic bacteria associated with necrotizing enterocolitis. All four exhibited bacteriostatic, though not bactericidal, activities against *Klebsiella aerogenes*, *Citrobacter freundii* and *Serratia marcescens*, but not *Escherichia coli*.

## 1. Introduction

Human milk (HM) feeding reduces risk for necrotizing enterocolitis (NEC), a severe inflammatory bowel disease that primarily affects preterm infants and occurs in the distal portion of the small intestine [1,2,3,4]. Part of the mechanism by which NEC risk may be reduced in infants fed HM is via bioactive peptides released from milk’s proteins during digestion [5,6]. Milk protein-derived bioactive peptides have an array of functions including antimicrobial, immunomodulatory, mucin-stimulatory, gut motility modulation, antioxidant and prebiotic [6,7,8,9,10]. As NEC is associated with inflammation and changes in the gut microbiome [11,12,13,14,15], the activities of milk peptides released in the gut could impact NEC risk. Peptidomics research has revealed that thousands of unique peptides are released from milk proteins during gastric [16,17] and intestinal [18] digestion in preterm infants, and some milk peptides survive to fecal excretion [19]. Among these peptides found in the infant gut, many have high homology with or are identical to known bioactive peptides, and novel antimicrobial peptides have been identified in these digesta [18,19]. Our previous finding that in vitro-digested colostrum and transitional HM (days two to eight postpartum) reduced lipopolysaccharide (LPS) and tumor necrosis factor (TNF)-induced inflammation and cytotoxicity in intestinal epithelial cells to a greater extent than undigested colostrum and transitional HM may be due to the bioactive peptides released during digestion [20]. Although microbiome research has revealed differences in overall microbial profiles between NEC and non-NEC infant stools [13], the specific microbes that may cause NEC remain [21] often associated with NEC, thus serving as good target pathogens for antimicrobial testing. Though many milk-derived peptides are antimicrobial, none have yet been tested for reduction of NEC incidence.

The objectives of this study were to: (1) characterize and compare peptides present in colostrum samples before and after exposure to an in vitro model of preterm infant digestion; (2) to predict antimicrobial activities of peptides using sequence-based prediction; and (3) to test antimicrobial activity of predicted peptides against neonatal pathogens. We hypothesized that colostrum digestion releases peptides with antibacterial action.

## 2. Materials and Methods

### 2.1. Colostrum Samples

Ten de-identified colostrum samples from different lactating parents of preterm infants were separated into two aliquots: (1) left undigested; and (2) digested separately using an in vitro digestion designed to simulate preterm infant digestion based on previous work [20,22]. In brief, the in vitro digestion protocol involved colostrum samples (200 µL colostrum + 2.2235 mL de-ionized water) being treated with pepsin (80 mg pepsin [≥250 units mg^−1^] in 2 mL of de-ionized water) and titrated to pH 4.0 with 1 M hydrochloric acid (gastric digestion) and shaken at 100 rpm 30 min at 37 °C. A variable amount (10–22 μL) of 1 M sodium bicarbonate was added to increase to intestinal pH of 6.0, followed by the addition of pancreatin and bile salts (20 mg pancreatin [4X USP] + 120 mg bile salts in 10 mL of 0.1 M sodium bicarbonate) and incubated at 37 °C with 100 rpm agitation for 30 min to simulate the initial stages of intestinal digestion. The pH was further increased to 7.0 followed by incubation for 2 h at 37 °C with 100 rpm agitation to simulate distal intestinal digestion [20,22]. After incubation, heat treatment at 90 °C for 15 min was applied to all samples to deactivate enzymes. All “undigested” colostrum aliquots were diluted with deionized water in the same volume of digestive additives as in the “digested” counterparts and exposed to the same final heat treatment.

### 2.2. Peptide Extraction from the Colostrum Samples

Peptides were extracted from undigested and digested colostrum samples using centrifugation, acid precipitation using trichloroacetic acid and solid phase extraction as described previously [16]. Briefly, all colostrum samples were centrifuged, and the liquid infranatant including proteins and peptides was collected. The collected samples were mixed with trichloroacetic acid to precipitate proteins, centrifuged and the supernatant including peptides was collected. The extracted peptides in both undigested and digested colostrum samples were further purified by solid phase extraction using C18 96-well plates (Glygen Corp, Columbia, MD, USA) and completely dried via vacuum centrifugation.

### 2.3. Peptide Analysis Using Liquid Chromatography and Mass Spectrometry (LC-MS)

Dried samples were reconstituted with 100 μL of 3% acetonitrile, 0.1% formic acid and diluted tenfold. Peptides were measured using an Orbitrap Fusion™ Lumos™ Tribrid™ mass spectrometer (Thermo Fisher Scientific, Waltham, MA, USA) combined with a NanoAcquity Ultra High-Performance Liquid Chromatography (UPLC) instrument (Waters Corporation, Milford, MA, USA). One microliter of peptides was loaded onto a C18 180 μm × 20 mm, 5-μm bead nanoAcquity UPLC trap column (Waters) and separated with a 100 μm × 100 mm, 1.7-μm bead Acquity UPLC Peptide BEH C18 column (Waters). Peptides were separated and eluted for 60 min at a flowrate of 0.5 µL min^−1^ using the following gradient conditions: 3% to 11.5% B, 0 min to 10 min; 11.5% to 20% B, 10 min to 31 min; 20% to 30% B, 31 min to 36 min; 30% to 95% B, 36 min to 45 min; 95% B, 45 min to 54.5 min, 95 to 3% B over 0.5 min then finally the column was re-equilibrated with 97% A for 5 min. Mobile phase A was 100% ultrapure water, 0.1% formic acid and mobile phase B was 100% acetonitrile, 0.1% formic acid. Peptides were ionized with a nanospray ion source with an electrospray voltage of 2320 V and an ion transfer tube temperature of 300 °C. MS spectra were acquired in positive ionization mode over an m/z range of 300–2000 with the Orbitrap at a resolution of 60,000. The automatic gain control (AGC) target was set to 4.0 × 10^5^, with a maximum injection time of 50 ms. The MS cycle time was set to 3 s. Precursor ions were automatically selected by the acquisition software for a data-dependent MS/MS scan based on the following criteria: ion-intensity threshold of 5.0 × 10^4^, charge state of 2–8 and exclusion duration of 60 s and mass tolerance of 10 ppm. The fragmentation of precursor ions was performed by electron-transfer/higher-energy collision dissociation (EThcD) with optimized electron-transfer dissociation (ETD) reaction times depending on charge state (2+, 130 ms; 3+, 70 ms; 4+, 50 ms; 5+, 40 ms; 6+ to 8+, 20 ms) and supplemental higher-energy collision-induced dissociation (HCD) activation (25% of collision energy). MS/MS spectra were acquired in the positive ionization mode over an m/z range of 300–2000 with the Orbitrap at a resolution of 30,000. The AGC target was set to 5.0 × 10^4^.

### 2.4. LC-MS Data Analysis

Peptide identification was performed using Thermo Proteome Discoverer (v2.2) with a SequestHT search engine from the LC-MS raw files based on database searching using an in-house HM protein sequence database (*n* = 378). Cleavage sites were set to “No-Enzyme (Unspecific)”. The precursor mass tolerance and fragment mass tolerance were set to 10 ppm and 0.5 Da, respectively. Allowed dynamic modifications included phosphorylation of serine and threonine, oxidation of methionine, acetylation at the N-terminus of the protein and amidation at the C-terminus of the protein. Peptide sequences of all proteins identified in digested colostrum samples that were not identified in undigested colostrum were examined to rule out porcine origin (digestive enzymes used for the in vitro digestion protocol were of porcine origin) using the Protein Basic Local Alignment Search Tool (BLASTp^®^) of the National Library of Medicine.

### 2.5. Homology Search against Known Bioactive Peptides

Identified HM peptides were examined for homology with known bioactive peptides using the Milk Bioactive Peptide Database (MBPDB; https://mbpdb.nws.oregonstate.edu/, accessed on 1 May 2024) [9]. The search parameters were set to “Sequence” for Search type, “80%” for Similarity threshold”, “Identity” for Scoring matrix and “human (homo sapiens)” for Species. 

### 2.6. Sequence-Based Prediction of Antimicrobial Peptides

The amino acid sequences of peptides found from digested samples were used as an input to AmPEP tool to predict their antimicrobial activity potential [23,24]. This tool is a sequence-based predictor, which recognizes distribution patterns of amino acid properties using a random forest algorithm to predict antimicrobial activity. Peptides with predicted probability values greater than 0.5 were classified as potentially antimicrobial. Four HM peptides with varying predicted antimicrobial probability values > 0.5 were chosen for empirical testing. Selecting peptides with varying predicted antimicrobial probabilities enabled a diverse and comprehensive assessment of potential antimicrobial activities.

### 2.7. Bacterial Strains and Culture Conditions

Bacteria commonly observed in neonates with culture-positive sepsis were used for antimicrobial testing [21,25,26,27,28]. *Citrobacter freundii* ATCC 13316 and *Serratia marcescens* subsp. *marcescens* Bizio were obtained from American Type Culture Collection (ATCC). *Klebsiella aerogenes* and *Escherichia coli* were obtained from Ward’s Science (strain not provided; Rochester, NY, USA). All bacterial strains were cultured on nutrient agar at 37 °C for 24–48 h. Single colonies were isolated for liquid culture. *E. coli* was grown in Luria-Bertani (LB) broth containing 1% (*w*/*v*) tryptone, 1% (*w*/*v*) sodium chloride and 0.5% (*w*/*v*) yeast extract; other strains were grown in nutrient broth containing 0.3% (*w*/*v*) beef extract and 0.5% (*w*/*v*) peptone. 

### 2.8. Antimicrobial Activity of Predicted Peptides

The top four candidate peptides (α_S1_-casein [157–163] QYVPFPP, α_S1_-casein [157–165] QYVPFPPFS, β-casein [153–159] VPQPIPQ and plasminogen [591–597] SWPWQVS) were synthesized by GenScript (Piscataway, NJ, USA) with ≥98% purity. Peptides were reconstituted with ultrapure water to 5 mg mL^−1^. Minimal inhibitory concentration (MIC) determination was performed based on published methods [29,30]. The antimicrobial assay was performed in a 96-well microtiter plate with two-fold dilution of each peptide ranging from 4.9 µg mL^−1^ to 2500 µg mL^−1^. Bacterial culture with a density equal to McFarland standard 0.5 was inoculated into each well in a 1:1 ratio with the peptide solution. Controls included a sterility control without inoculum and a growth control without peptides. MIC test with varying peptide concentrations of each peptide on bacterial growth was measured through readings at the optical density of 600 nm by a microplate reader at baseline and after incubation at 37 °C for 18–24 h. Promotion or inhibition on bacterial growth was identified by calculating the percentage change of optical density at 600 nm against the growth control. The data presented are the mean values obtained from two independent experiments. To test whether peptides had bactericidal effects, bacterial cultures from the wells that had been spiked with 2500 µg mL^−1^ of each peptide were spread on nutrient agar plates without peptides, and growth of bacteria was monitored over 48 h. 

### 2.9. Statistical Analysis

Peptidomic data were entered into SPSS (IBM SPSS Statistics for Windows, Version 25.0. IBM Corp., Armonk, NY, USA), and descriptive analyses were performed to assess normality. Peptide abundances were deemed non-normal; thus, a related-samples Wilcoxon Signed Rank test was conducted to compare peptide abundances between undigested and digested samples. Heat mapping was conducted via Heatmapper to evaluate differences of peptide profiles between undigested and digested colostrum samples [31].

## 3. Results

### 3.1. Comparison of Peptides Identified in Undigested and Digested Samples

**Figure 1:**
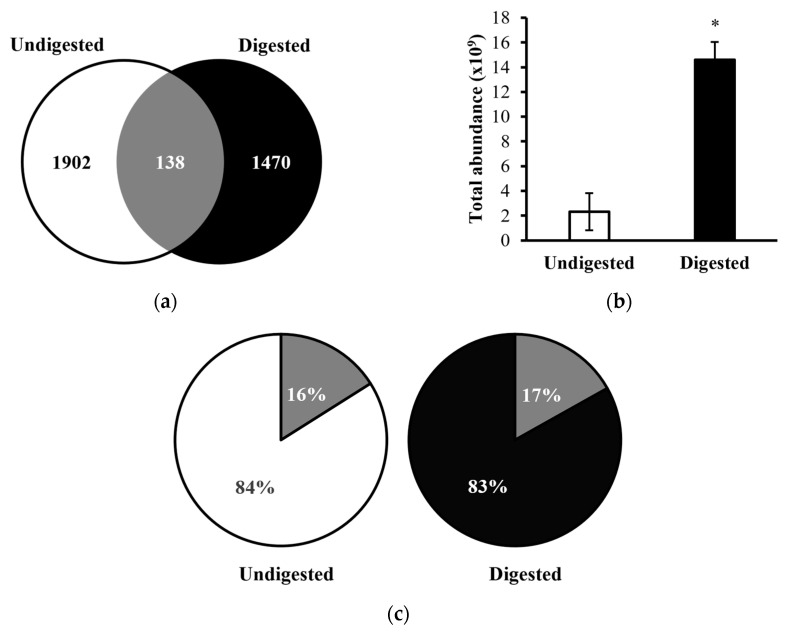
Venn diagram and bar graph of (**a**) counts; (**b**) total chromatographic abundances (Log10) of human milk peptides identified from undigested and digested human milk samples; (**c**) Pie charts show the percentage of the total abundance of common (16% and 17%, respectively, in dark grey) and unique (84% and 83%, respectively) proteins identified in undigested (in white) and digested (in black) samples. * *p* = 0.005; difference in total peptide abundance between undigested and digested using related-samples Wilcoxon Signed Rank test.

**Figure 2:**
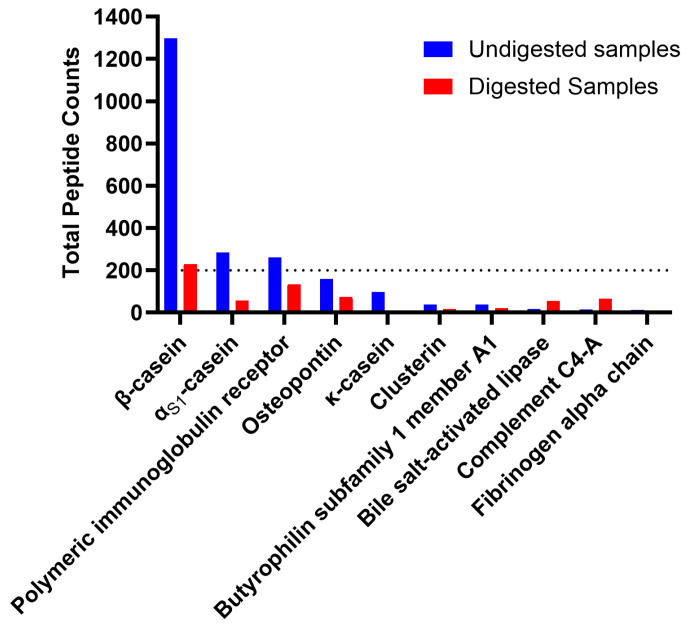
Top 10 parent proteins based on highest number of peptides that were derived in both undigested (blue) and digested (red) human milk samples.

**Figure 3:**
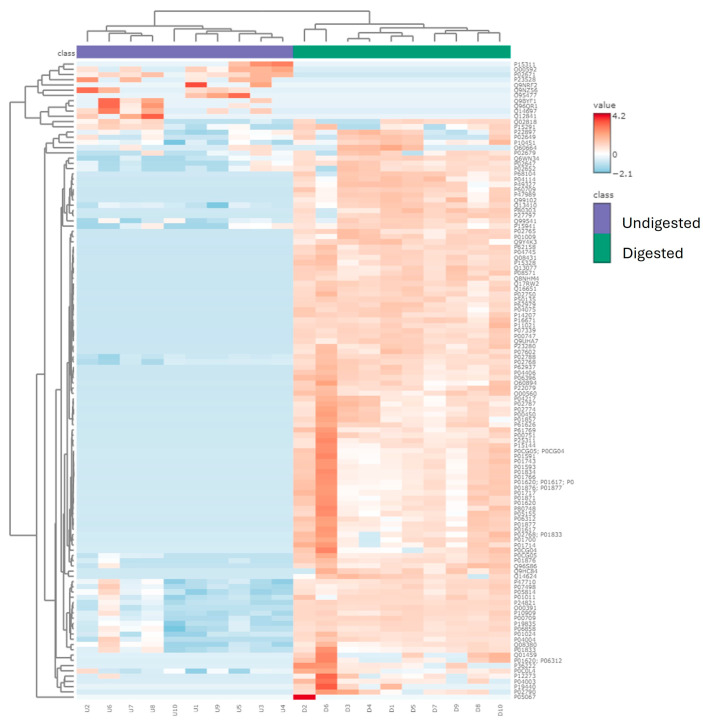
Heatmap and hierarchical clustering showing the similarity/differences of abundance pattern of human milk proteins that were identified using peptide profiles between undigested (U) and digested (D) samples.

A total of 2040 peptides were identified from the ten undigested colostrum samples, and a total of 1608 peptides were identified in the ten in vitro-digested colostrum samples (Figure 1a, Supplementary Tables S1 and S2). Among these peptides, 138 sequences were shared between the undigested and digested samples (making up 6.8% of peptides in the undigested samples and 8.6% of the peptides in digested samples by count). Total abundance of the in vitro-digested colostrum samples was significantly higher than the undigested samples (*p* = 0.005, nearly five times higher, Figure 1b). The 138 peptides present in both undigested and digested samples made up 16% of the total peptide abundance in the undigested samples and 17% of the total peptide abundance in the digested samples (Figure 1c). Among the top ten parent proteins from which peptides derived in the undigested and digested samples, β-casein, α_S1_-casein, polymeric immunoglobulin receptor, osteopontin, bile salt-activated lipase and complement C4-A were present in both undigested and digested samples (Figure 2). Heatmapping and hierarchical clustering showed a clear separation of the peptide profile patterns between undigested colostrum and in vitro-digested colostrum (Figure 3). 

Homology searching of all identified peptides against the Milk Bioactive Peptide Database [9] revealed 121 peptides in the undigested samples and 64 peptides in the digested samples that had over 80% homology to known bioactive peptides (Table 1; Supplementary Tables S3 and S4). Among identified peptide bioactivities from the undigested samples, the three most common functional groups were: angiotensin-converting enzyme (ACE)-inhibitory (35 peptides); antimicrobial (45 peptides); and increased cellular growth (52 peptides) (Table 1). The profile of peptide bioactivities in the digested samples differed from the undigested peptides: most bioactive peptides in the digested samples were ACE-inhibitory (43 peptides) and only one peptide in the digested samples had >80% homology with a known antimicrobial peptide (Table 1). 

### 3.2. Sequence-Based Prediction of Bioactive Peptides

The AmPEP algorithm classified 96 of the identified peptides in the digested samples as potentially antimicrobial, with a prediction probability range of 0.50–0.82. Four peptides (α_S1_-casein [157–163] QYVPFPP, α_S1_-casein [157–165] QYVPFPPFS, β-casein [153–159] VPQPIPQ and plasminogen [591–597] SWPWQVS) with varying predicted antimicrobial probabilities (0.55–0.73) were randomly selected for empirical testing. These four peptides were found in all 10 digested samples, but were not present in the undigested samples. Longer peptides containing these sequences from the same parent proteins were present in the undigested samples.

### 3.3. Antimicrobial Activity of the Peptides with Predicted Antimicrobial Activities

**Figure 4:**
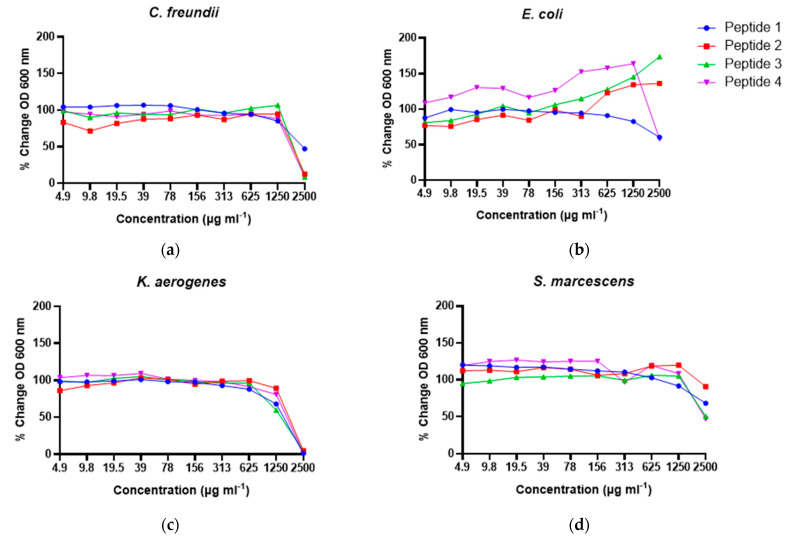
Percent change in optical density at 600 nm from the growth of common pathogenic bacteria incubated with synthesized peptides SP1-SP4. (**a**) *C. freundii*; (**b**) *E. coli*; (**c**) *K. aerogenes*; (**d**) *S. marcescens.*

The growth of *C. freundii* was inhibited by α_S1_-casein [157–165], β-casein [153–159] and plasminogen [591–597] at the MIC of 2500 µg mL^−1^ (Table 2). Though the MIC for α_S1_-casein [157–163] against *C. freundii* was >2500 µg mL^−1^ (Table 2), the 2500 µg mL^−1^ dose somewhat reduced growth (Figure 4a). The growth of the *E. coli* strain was not inhibited by any of the four peptides. However, a slight growth reduction of *E. coli* was observed when incubated with α_S1_-casein [157–163] and plasminogen [591–597] at concentrations of 1250 µg mL−1 and higher, whereas α_S1_-casein [157–165] and β-casein [153–159] enhanced growth at concentrations higher than 625 µg mL^−1^ (Figure 4b). All four peptides reduced the growth of *K. aerogenes* starting at 1250 µg mL^−1^ and completely inhibited its growth with an MIC of 2500 µg mL^−1^ (Figure 4c). The growth of *S. marcescens* was reduced to various extents when incubated with 2500 µg mL^−1^ of all four peptides (Figure 4d); however, the MIC was >2500 µg mL^−1^. In the bactericidal tests, growth of all the four bacterial strains were recovered in 24–48 h, indicating no bactericidal effects against *K. aerogenes*, *C. freundii* or *S. marcescens* for any of the tested peptides. This finding indicates the observed reductions in bacterial growth were due to a bacteriostatic effect.

## 4. Discussion

The relatively limited number of peptides that were similar between undigested and digested samples (138, making up 6.8% of undigested samples and 8.6% of digested samples) indicates that digestion caused at least partial degradation of most native milk peptides and the release of novel peptides from milk protein or native milk peptide precursors. Similarly, the limited percent of total abundance in the undigested (16%) and digested colostrum (17%) made up by peptides present in both sample types indicates that digestion induced major shifts in the overall peptide profile. The large increase in total peptide abundance from undigested to digested colostrum samples also demonstrates that digestion caused the release of a large amount of peptides from intact milk proteins. The heatmap also indicates that the abundance of peptides from each protein differs between undigested and digested colostrum samples. 

Homology search of the peptides identified against the MBPDB revealed 45 peptides in the undigested samples had >80% homology with a previously identified antimicrobial peptide, but only one peptide met that criterion in the digested samples. The lower number of known antimicrobial peptides in the intestinal digests compared with the colostrum cannot be understood to indicate that the intestinal digest peptides have less bioactive potential. Many peptides in the intestinal digest may be antimicrobial but have not been previously examined for this function. Therefore, we focused on testing the peptides present in the intestinal digests for novel antimicrobial action. Sequence-based prediction of antimicrobial activities yielded 96 peptides from the digested samples that were candidates for antimicrobial activity. From these, we selected four peptides (α_S1_-casein [157–163], α_S1_-casein [157–165], β-casein [153–159] and plasminogen [591–597]) with varying prediction scores to synthesize and test for antimicrobial action against four strains of bacteria previously observed in culture-positive blood samples of preterm infants in neonatal intensive care units with sepsis and/or NEC. Each of the four peptides inhibited *C. freundii*, *K. aerogenes* and *S. marcescens*, but only two peptides inhibited *E. coli*, and only to a small degree at the concentrations tested. The inhibitory effects of these peptides were overall strongest against *C. freundii* and *K. aerogenes*. The pathogen inhibitory effects of these peptides suggested a potential role of these digestion-released HM peptides in inhibiting the growth of pathogenic bacteria that are often associated with sepsis and NEC in premature infants. The results also indicate that MIC of the same peptide differed depending on the bacterial strain and that some peptides appeared to promote the growth of *E. coli*, similar to the findings from Beverly et al. [18]. Though all the tested peptides were able to inhibit microbial growth of *C. freundii*, *K. aerogenes* and *S. marcescens*, and two peptides inhibited *E. coli*, the MIC results did not differ based on the predictive scores, suggesting the need to refine the prediction tools. Though milk casein proteins are precursors for many known antimicrobial peptides, previous studies have not identified antimicrobial peptides from plasminogen. Therefore, the identification of plasminogen [591–597] as an antimicrobial peptide is particularly novel. Plasminogen and plasmin are abundant in HM [32]. The antimicrobial activities of the four peptides—α_S1_-casein [157–163] (QYVPFPP), α_S1_-casein [157–165] (QYVPFPPFS), β-casein [153–159] (VPQPIPQ) and plasminogen [591–597] (SWPWQVS)—are closely tied to their sequences and structural properties. The α_S1_-casein peptides possess amphipathic structures with a balance of hydrophobic, aromatic and polar residues, which enhance their ability to interact with and disrupt bacterial membranes. The presence of proline residues in α_S1_-casein [157–163] and [157–165] introduces kinks that increase flexibility and membrane interaction. β-casein [153–159], with its proline-rich sequence, also displays structural flexibility, allowing it to interact effectively with bacterial membranes through hydrophobic and polar residues. Plasminogen [591–597] features aromatic and hydrophobic residues that facilitate membrane disruption, with serine and tryptophan residues enabling both hydrophilic and hydrophobic interactions. These structural features—hydrophobicity, aromaticity and flexibility—play crucial roles in the peptides’ ability to target and disrupt bacterial membranes, thereby exhibiting antimicrobial activity.

The four peptides selected for antimicrobial testing were present in all ten HM samples. Additionally, the MICs are relatively higher than concentrations that would likely be present within the infant intestines; thus, therapeutic dosages of these peptides would likely need to be delivered to exert potential in vivo effects. We suspect that milk peptides may be supporting microbiome development in the neonate by reducing potentially pathogenic bacteria while promoting commensal bacteria. Future work should examine whether these peptides can increase the growth of beneficial infant gut microbes, such as Bifidobacterium, as previously reported [18,33]. HM antimicrobial peptides likely represent one component of a system of milk components (e.g., milk oligosaccharides [34,35,36,37,38], immunoglobulins [36,39,40,41]) that help guide infant gut microbial development. 

## 5. Conclusions

By combining peptidomic analyses of digestion-released peptides from human colostrum with a machine learning-based modeling tool followed by synthesis and testing of predicted antimicrobial peptides, we were able to identify four peptides that can suppress certain common pathogenic bacteria that are associated with NEC and sepsis in the premature infant. Our findings provide insights into the potential capacity of HM peptides to inhibit the growth of pathogenic virulent bacteria in the infant gut and exert potential protection against infection.

## Figures and Tables

**Table 1:** Summary of the Milk Bioactive Peptide Database (MBPDB) >80% homology search results.

Counts of Peptides Identified by MBPDB Search	Undigested	Digested
Total	152	64
ACE-inhibitory	35	43
Anticancer	-	2
Antimicrobial	45	1
Antioxidant	1	4
DPP-IV Inhibitory	-	1
Immunomodulatory	19	2
Increase cellular growth	52	9
Opioid	-	2

**Table 2:** Minimum inhibitory concentrations (MICs) and predicted probability of antimicrobial peptides (AMP) of synthesized peptides against common pathogenic bacterial strains, with MIC represented in units of µg mL^−1^.

			*C. freundii*	*E. coli*	*K. aerogenes*	*S. marcescens*
Peptide ID	Sequence	Predicted Probability of AMPs	MIC	MIC	MIC	MIC
α_S1_-casein [157–163]	QYVPFPP	0.67	>2500	>2500	2500	>2500
α_S1_-casein [157–165]	QYVPFPPFS	0.73	2500	>2500	2500	>2500
β-casein [153–159]	VPQPIPQ	0.61	2500	>2500	2500	>2500
plasminogen [591–597]	SWPWQVS	0.55	2500	>2500	2500	>2500

## Data Availability

Data available upon request.

## Supplementary Materials

The following supporting information can be downloaded at: https://www.mdpi.com/article/10.3390/nu16132040/s1, Table S1: List of the peptides identified from the undigested human milk samples; Table S2: List of the peptides identified from the digested human milk samples; Table S3: MBPDB search results of the peptides identified from the undigested human milk samples; Table S4: MBPDB search results of the peptides identified from the digested human milk samples.
